# Valorization of Bioactive Compounds from Lingonberry Pomace and Grape Pomace with Antidiabetic Potential

**DOI:** 10.3390/molecules29225443

**Published:** 2024-11-18

**Authors:** Elena Neagu, Gabriela Paun, Camelia Albu, Gabriel Lucian Radu

**Affiliations:** Centre of Bioanalysis, National Institute of Research and Development for Biological Sciences, 296 Splaiul Independentei, 060031 Bucharest, Romania; elena.neagu@incdsb.ro (E.N.); camelia.albu@incdsb.ro (C.A.)

**Keywords:** bioactive compounds, by-products, lingonberry pomace, grape pomace, antidiabetic potential

## Abstract

In recent years, increased attention has been paid to the recovery of bioactive compounds from waste and by-products resulting from the agro-industrial sector and their valorization into new products, which can be used in the health, food, or agricultural industry, as innovative and sustainable approaches to waste management. In this work, two of these by-products resulting from the fruit-processing industry were used for the recovery of bioactive compounds (polyphenols), namely lingonberry pomace (*Vaccinium vitis-idaea*) and grape pomace (*Vitis vinifera*). Two green extraction techniques were employed to obtain hydroalcoholic extracts (solvent: 50% EtOH, 10% mass): ultrasound-assisted extraction (UAE) and accelerated solvent extraction (ASE). The extracts were subjected to micro- and ultrafiltration processes, and further analyzed to determine the bioactive compound content through spectrophotometric (UV-Vis) and chromatographic (HPLC-PDA) methods. Additionally, the extracts exhibited significant enzyme inhibition, particularly against α-amylase and β-glucosidase, suggesting potential anti-diabetic properties. The extracts characteristics, polyphenolic content, antioxidant capacity and enzyme inhibitory ability, were statistically compared, and significant differences were found between the two extraction methods. The grape pomace concentrated extracts showed a pronounced inhibitory activity on both analyzed enzymes compared to the lingonberry pomace concentrated extracts, closer to the standard used; e.g., IC_50_ α-amylase = 0.30 ± 0.01 µg/mL (IC_50_ acarbose = 0.3 ± 0.01 µg/mL), IC_50_ α-glucosidase = 0.60 ± 0.01 µg/mL (IC_50_ acarbose = 0.57 ± 0.02 µg/mL). These findings highlight the potential of agro-industrial residues as bioactive compound resources, with their valorization through application in food, nutraceutical, or pharmaceutical industries therefore contributing to the sustainable development and promotion of circular economy principles with the recovery of valuable inputs from plant by-products.

## 1. Introduction

Recently, obtaining bioactive compounds from natural sources has become increasingly attractive. Bioactive compounds are recognized for their antioxidant, antibacterial, and anti-inflammatory potential [1]. Natural compounds are preferred because they have reduced side effects and cost less than most synthetic compounds.

The fruit processing industry generates large amounts of residues/by-products, leading to significant environmental problems if they are not managed effectively [2]. An efficient way of using these by-products in the medium and long term is their reuse as raw material for the recovery of bioactive compounds and obtaining new products with added value and high benefits for health. This approach is aligned with the current principle of the circular economy [3]. Pomace contains significant quantities of phenolic compounds, dietary fiber, polysaccharides, enzymes, antioxidants, and has many nutrients and health benefits [4]. These compounds are of great interest for functional food production, medicines, and antioxidants with applicability in the cosmetic, food, and/or pharmaceutical industries [5].

*Vaccinium vitis-idaea* (lingonberry) (genus *Vaccinium*, family Ericaceae) have been used in folk medicine, in Asian and European countries, for treatment of urinary tract infections, gastrointestinal disorders, neurodegenerative diseases, and inflammatory conditions associated with the presence of free radicals [6,7]. In Chinese folk medicine, lingonberry has been successfully used in cases of inflammation, obesity, neurodegenerative diseases, in brain-aging treatment, and in prevention actions [8,9].

Lingonberries grow in natural habitats but are also cultivated for commercial purposes in the Scandinavian countries and Canada, where they are consumed either fresh or frozen or processed into food products and pharmaceutical preparations [10]. Considerable amounts of by-products considered residues are generated during processing, causing the loss of bioactive substances and valuable nutrients [11]. For example, in Sweden, approximately 140 tons of pomace lingonberries are generated annually [12].

*V. vitis-idaea* fruits contain numerous nutrients such as multivitamins, polysaccharides, dietary fiber, and minerals, bioactive compounds such as anthocyanins, proanthocyanidins, flavonols, phenolic acids, simple phenolics, phytosterols [13,14], hydroxycinnamic acids, triterpenoids, and flavonoids, functional components with beneficial effects for health [15,16]. Lingonberry has strong anti-inflammatory, antioxidant, antithrombotic, hypoglycemic, antiseptic, and antibacterial properties [17,18,19].

Grape pomace is the predominant by-product resulting from the processing of grapes, especially in the wine industry, containing seeds, stems, and skins as main components, and representing approximately 25% of the processed grapes [20]. Grape pomace represents an important source of bioactive compounds (approximately 70% of bioactive compounds remain after processing in pomace), such as polyphenols, flavonoids, dietary fibers [21], organic acids, reducing sugars, pigments, fatty acids, as well as significant amounts of anthocyanidins [22].

These compounds are responsible for the antioxidant, anti-inflammatory, anti-obesity [23], antibacterial, and anticancer activities of gooseberry [24,25,26]. Polyphenolic compounds are free radical scavengers, scavengers of harmful products of aerobic metabolism that lead to oxidative stress in the body. Oxidative stress is associated with the occurrence of numerous diseases such as those cardiovascular, of and neurological disorders, cancer, and disorders related to aging. Numerous in vitro, in vivo, and epidemiological studies have revealed that polyphenols are of great importance in the prophylaxis and treatment of these conditions through their remarkable antioxidant activity [27,28,29].

Polyphenols have potential applications also in the food and beverage industry, as preservatives and natural dyes [30]. Grape pomace contains large amounts of anthocyanins and anthocyanidins in the skins because only 30–40% of the anthocyanins contained in the grapes are extracted during vinification [30]. These compounds show enhanced antioxidant, anti-inflammatory, and antimicrobial capacity [31,32]. Anthocyanins also have applications in the food, cosmetic, and pharmaceutical industries [20].

Grape pomace is also an important source of dietary fiber, which is useful in maintaining the health of the digestive system and decreasing the risk of diabetes, chronic conditions, and heart disease [20]. Dietary fibers can also be useful in the food industry to obtain functional foods with a prebiotic and hypocholesterolemic effect [33].

Numerous methods have been used to extract bioactive compounds, especially polyphenolic compounds, from plant sources. These methods include traditional methods such as maceration, Soxhlet extraction, and hydrodistillation, as well as modern, ecological methods: assisted extraction ultrasound (US), microwave-assisted extraction (MAE), enzyme-assisted extraction (EAE), supercritical fluid extraction, and accelerated solvent extraction (ASE) [34,35].

In this study, two of these new, ecological methods, widely recognized as “green” and friendly to the environment were used: ultrasound-assisted extraction (UAE) and accelerated solvent extraction (ASE). These methods usually involve low operating and maintenance costs, moderate energy consumption, low working temperatures and processing time, low amounts of water and solvents, low environmental impacts, and obtaining high-quality extracts [36].

Ultrasound-assisted extraction (UAE) is based on the application of an ultrasound that generates a cavitation phenomenon [37]. The action of cavitation, and implicitly the extract quality of the sonication, are influenced by the frequency, power, time, and amplitude [1,34,38].

Accelerated Solvent Extraction (ASE) is based on the usage of high pressure, which allows the solvents to be kept above their boiling point, which makes the extraction of the target compound easier (by improving mass transfer and diffusion phenomena), obtaining an increased efficiency of the extraction process [39].

Micro-, ultra- and nanofiltration membrane technologies represent a viable alternative to traditional technologies. They are considered green processing technologies that allow the purification, fractionation, and concentration of polyphenols without requiring high temperatures that negatively affect their functionality [40].

Diabetes mellitus–a metabolic disease whose incidence has tripled in the last 20 years among adults [41] due to sedentary lifestyles, obesity, and unhealthy eating habits, requires serious and early management to avoid complications or to slow down complications, including nephropathy, cardiopathy, retinopathy, diabetic foot [42].

Diabetes can be controlled by oral medications or insulin administration. Oral antidiabetics reduce insulin resistance (e.g., thiazolidinediones, metformin) or inhibit the enzymes involved in carbohydrate digestion (e.g., acarbose) [43]. Unfortunately, oral antidiabetics (e.g., acarbose) also have some unwanted effects like discomfort, flatulence, diarrhea, cardiovascular diseases, weight gain, and hepatotoxicity [43]. In addition, they are very expensive and difficult to access in developing countries [44]. Obtaining antidiabetic agents from natural products (rich in polyphenolic compounds) represents an alternative, due to their availability, good efficiency, and low toxicity [45]. Scientific data have shown that polyphenolic compounds have anti-diabetic potential through their anti-hyperglycemic effect, being useful in the prophylaxis and treatment of diabetes and its complications [46,47].

The beneficial effects of polyphenolic compounds are conditioned by their availability and accessibility. A small part of the ingested polyphenol compounds is absorbed in the small intestine [48], the rest being the result of the action of the intestinal microbiota and microbial enzymes, resulting in bioaccessible phenolic metabolites, different from the initial compounds [49]. Recent studies have shown that gut microbiota dysbiosis is closely related to the onset and development of diabetes [50] because the intestinal microbiota interacts with endocrine functions, homeostasis of carbohydrates and lipids, immunity, and systemic inflammation [51]. Thus, targeting the gut microbiota may be another approach to managing T2DM [52].

The use of bioactive compounds with an inhibitory effect on digestive enzymes can be an alternative to synthetic drugs on the market, avoiding the complications they cause [53].

In this context, the present study tested the antidiabetic potential of some hydroalcoholic extracts from lingonberry residues and grape pomace, enriched in polyphenolic compounds by analyzing their inhibitory capacity on α-amylase and α-glucosidase, enzymes involved in the digestion of carbohydrates. We chose these sources because there is not enough data to show the antidiabetic potential of grape pomace and lingonberry residues by inhibiting digestive enzymes. In addition, we used micro- and ultrafiltration membrane technologies to obtain extracts enriched with bioactive compounds.

## 2. Results and Discussions

### 2.1. Total Phenolic and Total Flavonoid Contents (TPC and TFC)

Table 1 presents the TPC and TFC values found in the extracts obtained by UAE and ASE, using spectrophotometric methods.

From the data presented in Table 1, it can be noted that the ASE method is more efficient than UAE, obtaining higher values for bioactive compound content, for both polyphenols (increase of 1.4 times) and flavones (an increase of 1.5–1.7 times) in the case of lingonberry pomace extracts. We also obtained higher TPC and TFC values for the concentrated extract (2440.62 ± 14.56 CAE µg/mL and 495.65 ± 22.52 RE µg/mL, respectively) compared to the microfiltrate extract (1804.37 ± 24.36 CAE µg/mL and 336.25 ± 12.31 RE µg/mL, respectively). The ASE method uses solvents at high pressures and temperatures, which accelerates the kinetics of the extraction process, resulting in a faster and safer process. Water and ethanol are the most used solvents that can extract different classes of compounds, such as polyphenols, but some conditions, such as high temperatures and extraction times, can lead to their degradation and the loss of their bioactivity. Therefore, in our study, the extraction was performed at 60 °C in three short cycles of 15 min [54].

As compared to other studies’ data for berries, our results for lingonberries were superior to those presented for blueberries (305.4 mg GAE/100 g) and red raspberries (357.80 mg GAE/100 g) [55,56].

For grape pomace extracts, the UAE method was more efficient, obtaining an increase of 1.66–1.82 times for polyphenols and 1.7 for flavones. UAE is an innovative extraction technique that uses ultrasound waves inducing cavitation and pressure variations through the extracting solvent, leading to the degradation of the cell wall and, consequently, improving the phytocompounds release into the extraction solvent. UAE extracted 87% antioxidants from grape pomace compared to 22% antioxidants extracted by maceration [57].

Moreover, UAE helps to improve the extraction process at low temperatures, causing minimal degradation of the molecular characteristics of the phytocompounds in plant materials, and has other advantages over conventional extraction procedures, including a shorter time, less use of solvents, a higher extraction yield, and a lower operating costs [58,59].

The TPC and TFC results were higher in concentrated extracts of grape pomace (1041.12 ± 26.25 CAE µg/mL and 124.40 ± 5.62 RE µg/mL, respectively) compared with the microfiltrate extracts (744.67 ± 15.32 CAE µg/mL and 81.20 ± 2.58 RE µg/mL, respectively). The obtained results revealed that the microfiltration and concentration processes influence the content of phenolic compounds in the investigated by-product extracts.

Numerous studies have proved that UAE shows superior results when compared to conventional extraction methods; e.g., Romanini et al. also obtained higher contents of polyphenols, anthocyanins, and flavonoids compared with traditional extraction methods for grape pomace residues [55].

### 2.2. High-Performance Liquid Chromatography with Photodiode Array Detector (Hplc-Pda) Analysis of Individual Phenolic Compounds

**Figure 1:**
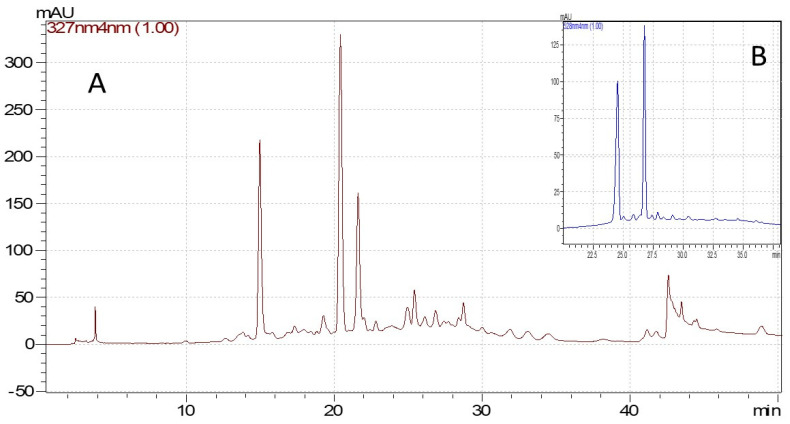
Chromatograms for lingonberry residue’s concentrated extract, ASE method ((**A**)—the phenolic acids and flavonoids chromatogram at 327 nm, (**B**)—the anthocyanidins and anthocyanins chromatogram at 528 nm).

**Figure 2:**
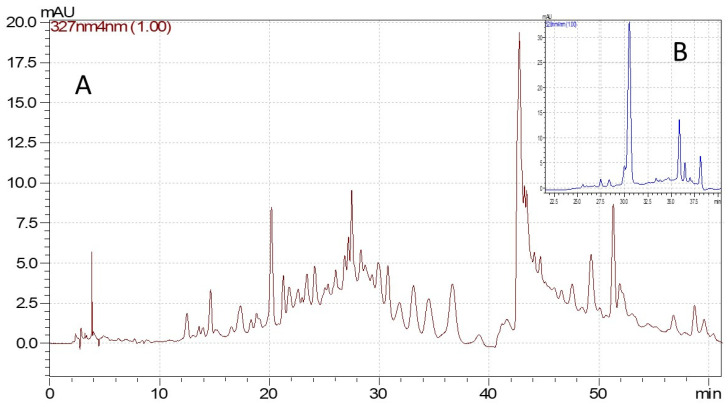
Chromatograms for grape pomace extract, ASE concentrate ((**A**)—phenolic acids and flavonoids chromatogram at 327 nm, (**B**)—anthocyanidins and anthocyanins chromatogram at 528 nm).

The phytochemical compounds found in the investigated extracts were identified using the HPLC-DAD technique and reported in Table 2 and Table 3, and Figure 1 and Figure 2. The chromatographic method was validated in-house and presented good linearity (R^2^ ≥ 0.999) in the concentration range 0.1–50 µg/mL [60]. The individual phenolic compounds were identified based on their retention time and UV spectra compared with certified reference materials.

As a result of the HPLC analysis around 21 polyphenolic compounds were identified and quantified in the extracts obtained from lingonberry and grape residues (Table 2). The observation regarding the differences between microfiltrated and concentrated extracts also applies to the HPLC analysis results. The UAE and ASE concentrated extracts contain high amounts of catechin (279.39 ± 18.6 μg/mL and 331.66 ± 15.63 μg/mL, respectively), chlorogenic acid (71.79 ± 4.89 μg/mL and 89.09 ± 4.65 μg/mL, respectively), and epicatechin (29.35 ± 1.32 μg/mL and 43.39 ± 2.56 μg/mL, respectively.

A statistically significant (*p* < 0.05) increase in the extractability of individual phenolic compounds from microfiltrated UAE and ASE extracts was observed for all compounds listed in Table 2 with the exception of chlorogenic acid, epicatechin, and myricetin. For concentrated extracts, increased extractability was achieved for all the phenolic compounds.

Analyzing the results obtained for anthocyanins, as presented in Table 2, a remarkable content of delphinidin-3-glucopyranoside (84.57 ± 3.56 μg/mL) and cyanidin-3-glucopyranoside (74.56 ± 2.89 μg/mL) was obtained, which was higher in the concentrated extracts obtained by UAE method.

In the literature, anthocyanins have been described as the main compounds of *Vaccinium* fruits, representing 78–81% of the total individual phenolic content. Spinola et al. reported delphinidin-O-hexoside as the predominant compound (31.80–32.42%), followed by petunidin-O-hexoside (18.09%) and malvidin-O-hexoside (12.54%) in *V. cylin-draceum* fruits, while different percentages were quantified in *V. padifolium*, namely the following: delphinidin-O-hexoside (15.18%), petunidin-O-hexoside (11.05%) and malvidin-O-hexoside (10.46%) [61]. Other studies have described, as the main anthocyanins for *Vaccinium* species, malvidin-3-O-glucoside, delphinidin-3-O-galactoside, and petunidin-3-O-glucoside compounds [62,63], while cyanidin glycosides were the main polyphenols found in *V. vitis-idea* and *V. meridionale* Swartz berries [64,65]. The study of Xu et al. highlighted the content of catechin as over 60% of the total tannin content (0.52–4.13 mg/g DW) and the cyanidin-3-O-galactoside (0.48–4.17 mg/g DW) as the predominant component present in lingonberries extracts [17].

In other studies, anthocyanins, flavanols, benzoic acid derivatives, epicatechin, and catechin were identified as the major components of lingonberry extracts [66,67].

In the case of grape pomace extracts, the HPLC results are presented in Table 3 and Figure 2. The obtained values indicate very small differences between the two extraction methods regarding the bioactive compound profile and content. It can also be observed that the concentrated extracts present higher amounts of polyphenolic compounds compared to the initial extracts, which demonstrates the efficiency of the ultrafiltration processes. Except for the four phenolic compounds, rutin, ellagic acid, quercetin 3-β-D-glucoside and quercitrin; all the other ten polyphenols were more abundant in the ASE extracts, especially the concentrated ones. Epicatechin was the polyphenolic compound with the highest concentration determined, 22.59 ± 1.35 μg/mL in ASE extract and 13.97 ± 0.29 μg/mL in UAE extract, respectively). A possible explanation for these high levels observed for (−)-epicatechin as compared to those of (+)-catechin is the hydrolysis of the galloylated precursors, such as (−)-epicatechin gallate.

However, the quantification of anthocyanins and anthocyanidins in grape pomace extracts reported a maximum amount also in the ASE extracts, with malvidin-3-glucoside being the only compound with an increased amount in the UAE extracts (19.79 ± 0.89 μg/mL in the initial extract and 21.76 ± 1.56 μg/mL in the concentrated one). Other representative flavonoids were: (+)-catechin (13.35 ± 0.56 μg/mL), quercitrin (12.88 ± 0.89 μg/mL), quercetin 3-β-D-glucoside (6.25 ± 0.34 μg/mL) and gallic acid (4.96 ± 0.02 μg/mL).

Cisneros-Yupanqui et al. obtained (+)-catechin (407.87  ±  0.13 mg/kg) and epicatechin (363.62  ±  0.61 mg/kg) as the predominant compounds in grape pomace (both white and red varieties, with higher amounts in the white ones) [68]. In other studies, epicatechin was the predominant compound in red currants and catechin in red currants from Cabernet Sauvignon and Feteasca Neagra grapes [53,69].

Another recent study on grape pomace reported quercetin as the main flavonol (56.66 mg/100 g DW) in the “Merlot” variety, and gallic acid as principal phenolic acid (18.69 mg/100 g DW) in the “Bordeaux” variety [4].

### 2.3. Determination of Antioxidant Activity

In order to test the in vitro antioxidant activity of the extracts, two common antioxidant assays were used: 2,2-Diphenyl-1-picrylhydrazyl assay (DPPH) and the ferric-reducing antioxidant power (FRAP). Antioxidant activity can be monitored by a variety of assays with different mechanisms; therefore, the selection of methods for valid evaluation of antioxidant potential depends a lot on the variety of compounds from different matrices [66]. The results expressed as IC_50_ (µg/mL) and EC_50_ (µg/mL) are presented in Table 4.

The antioxidant activity determined by the DPPH method indicated that the majority of the extracts show antioxidant activity close to that of the standard used, vitamin C, with the concentrated extracts having even more pronounced antioxidant activity than the standard. The concentrated extracts have a higher antioxidant activity than the initial extracts due to the increased content of polyphenolic compounds, as a result of ultrafiltration processes.

In our study, the concentrated grape pomace extracts had a higher antioxidant activity than the concentrated lingonberries pomace extracts. The pronounced antioxidant activity of the concentrated extracts, and especially of those obtained by ASE (IC_50_: 0.30 ± 0.01 µg/mL for grape pomace, IC_50_: 0.69 ± 0.02 µg/mL for lingonberry pomace) can be attributed to the different composition of polyphenolic compounds. The high content of catechin and epicatechin in grape pomace extracts could determine the high antioxidant activity of these extracts, with these compounds being known for their antioxidant properties [70].

The results for reducing power activity indicate a lower activity than the standard compound for most extracts, regardless of the method used to obtain the extracts. The reducing power is higher for the concentrated extracts compared to the initial ones (microfiltered); the concentrated grape pomace extracts obtained by ASE showed antioxidant activity higher than the standard, EC_50_ = 39.39 ± 1.56 µg/mL, compared to EC_vitamin C_ = 42.47 ± 1.32 µg/mL.

The increased amount of free radicals due to oxidative stress is also associated with the onset of diabetes and its complications [71,72]. Exogenous antiradical compounds can reduce the oxidative stress involved in this condition [73].

Numerous studies reported a high antioxidant potential, both in an in vitro and in vivo environment, when using grape pomace extracts [74,75]. Also, many studies have shown that red grape pomace contains higher amounts of polyphenols and flavonoids; therefore, its antioxidant activity is much higher compared with that of white grape pomace [22].

The lingonberry pomace extract antioxidant capacity is superior to that of other berries, which allows it to serve as a high-quality functional food [7,13,75].

A study carried out on hydroalcoholic and ethyl acetic extracts of lingonberries from central Poland showed strong antioxidant activity assigned to the great content of flavonoid, polyphenolic compounds, and anthocyanins in the investigated extracts [66].

### 2.4. Antidiabetic Potential Testing-α-Amylase and α-Glucosidase Inhibition Activity

The results obtained regarding inhibitory capacity of lingonberry pomace and grape pomace extracts on α-amylase and α-glucosidase enzymes are presented in Table 5. To highlight the influence of the polyphenols and flavones content on the extracts’ ability to inhibit the enzymes taken in the study, these parameters were analyzed by Student’s *t*-test and the determined data were statistically compared, *p* < 0.05.

The concentrated lingonberry extracts obtained by both extraction methods showed moderate inhibitory activity on both enzymes, lower than the standard (Table 5). The extracts obtained by UAE presented higher inhibitory activity than those obtained by ASE.

In a study carried out on lingonberry extracts from two species of *Vaccinium* endemic to Portugal, *V. cylindraceum* (Azores blueberry) and *V. padifolium* (Madeira blueberry), the extracts showed effective inhibitory capacity on glucosidase and moderate inhibitory capacity on amylase compared to the standards used [61]. Anthocyanins were the majority of compounds determined in fruits and there was a correlation between the content of polyphenolic compounds and the reported bioactivities [61].

Studies carried out on several species of *Vaccinium*, including *V. ashei* Reade, *V. corymbosum* L., and their hybrids, revealed high antioxidant activity and high inhibitory activity on α-glucosidase, correlated with large amounts of anthocyanins and polyphenolic compounds determined mostly in skins rather than in pulp [72].

In another study, methanolic extracts of red currant, obtained by UAE, presented the highest reported result (almost 94%) of α-amylase inhibition, and catechin and procyanidin B2 were the most representative polyphenolic compounds probably responsible for this effect [76]. A total of 95% hydroalcoholic extracts from red lingonberry, obtained by UAE, presented higher concentration of polyphenolic compounds and almost 90% inhibition of α-glucosidase [53,77].

Studies have shown that polyphenolic compounds such as flavan-3-ols (quercetin), anthocyanins, and phenolic acids—such as ferulic, chlorogenic and caffeic acids—can improve blood glucose levels by decreasing ROS levels, inflammation, and protein glycation, inhibiting important enzymes related to T2DM and carbohydrate metabolism [78].

The concentrated grape pomace extracts exposed pronounced inhibitory activity on both analyzed enzymes, α-amylase and α-glucosidase, close to that of the standard used, acarbose (Table 5). The extracts obtained by ASE showed higher inhibitory activity than those obtained by UAE. The pronounced inhibitory activity can be correlated with the high content of catechin and epicatechin in the grape pomace extracts, these compounds being known for their antioxidant but also antidiabetic and antiobesity activity [23].

In many studies, grape pomace has been identified as an α-glucosidase and α-amylase inhibitor, showing a potential therapeutic action in the management of diabetes [79,80,81,82].

White grape pomace extracts showed an inhibitory effect on α-amylase (IC_50_ = 56.45 ± 0.05 mg/mL) as well as on α-glucosidase, stronger than that of red gooseberry (IC_50_ = 154.46 ± 0.82 μg/mL) [77]. Phenolic compounds from different food sources have been reported to be responsible for α-amylase inhibition [83].

In other studies, grape residues showed inhibitory activity on the two enzymes studied here, superior to the acarbose used as a standard. Thus, in a study carried out by Kong, 2018 [84], the aqueous extract of Chardonnay grape seeds indicated a higher in vitro inhibition activity of α-glucosidase than acarbose: IC_50_ = 25.25 ± 0.53 μg/mL and α-amylase: IC_50_ = 66.68 ± 1.1 g/mL; the inhibitory effects were stronger than those of acarbose and the predominant compounds in the extracts were catechin and epicatechin, 44.12 ± 0.21 mg/mL, and, respectively, 111.23 ± 1.29 mg/g [84].

The use of these target enzymes, α-amylase and α-glucosidase, in this context, is a relatively new approach, which has stood out in recent years [77]. Recent studies on polyphenolic extracts have shown the potential of polyphenolic compounds in the control of diabetes. Such attempts are based on some phenolic compounds’ capacity to inhibit digestive enzymes, through carbohydrate digestion leading to the slowing down of the increase in blood sugar [43,84]. Some characteristics of polyphenolic compounds such as molecular weight, number, and substitution positions make them suitable for inhibiting digestive enzymes [79].

Given the huge potential of the by-products resulting from the agri-food sector, it is necessary to develop methods for their reuse and recovery of their bioactive compounds through valorization of the potential to obtain functional foods, cosmetic products, food processing or obtaining supplements with therapeutic uses.

## 3. Materials and Methods

### 3.1. Chemicals and Reagents

Standards for caffeic acid, (+)-catechin, chlorogenic acid, cyanidin, p-coumaric acid, formic acid, isorhamnetin, luteolin, quercitrin, quercetin 3-glucoside, quercetin, rutin, kaempferol, and vitamin C were obtained from Sigma Aldrich (Darmstadt, Germany). Gallic acid, (−)-epicatechin, ellagic acid, and myricetin were purchase from Fluka (Buchs, Switzerland). From Roth (Carl Roth GmbH, Karlsruhe, Germany), delphinidin, peonidin-3-glucoside, and malvidin were acquired, while petunidin-3-glucoside was purchased from PhytoLab (Dutendorfer, Germany). Cyanidin-3-glucoside, malvidin-3-glucoside, and delphinidin-3-glucoside were obtained from Polyphenols AS (Hanaveien, Norway). HPLC-grade reagents (methanol, ethanol, acetic acid and acetonitrile) were obtained from Riedel-de Haen (GmbH, Seelze, Germany).

### 3.2. Sample Preparation and Extraction Protocols for Phenolic Compounds

Samples of grape and lingonberry pomace obtained after processing the raw material were purchased from a small fruit juice-producing company from Focsani (Lorelu Serv S.R.L.) (Vrancea County, Romania). The pomace was stored at −20 °C until processing. Then, the pomace samples were subjected to a drying process in a convection oven (POL-EKO, SWL 115, Poland) at 40 °C for 48 h. The temperature was selected so that there was a minimum loss of heat-sensitive nutrients. After 48 h, the moisture content of the pomace was calculated as 10% wet basis and the dried material was ground into a fine powder with particle size < 300 µm using a Grindomix GM100 mill (Retsch, Haan, Germany).

To obtain the extracts, two modern, environmentally friendly methods were used: ultrasound-assisted extraction (UAE) and accelerated solvent extraction (ASE). The extraction conditions used in the experiments (including the sample concentration) were chosen based on the results obtained in previous research [85] when the optimal conditions for high-yield polyphenol extraction were studied.

#### 3.2.1. Ultrasound-Assisted Extraction (UAE)

The UAE process was performed using an ultrasonic bath (model Transsonic T460 H, Elma, Germany) at a working frequency of 35 kHz. The ground plant material was mixed with aqueous ethanol solution 50% (*v*/*v*) with stirring for 1 h at room temperature, then it was placed in the ultrasonic bath for 1 h and finally filtered through Whatman filter paper (Sigma-Aldrich; Merck KGaA, Darmstadt, Germany) and stored at 4 °C for further analysis.; the temperature in the ultrasonic bath was maintained below 60 °C to avoid compounds’ degradation.

#### 3.2.2. Accelerated Solvent Extraction (ASE)

The extraction was carried out by means of an accelerated solvent extractor Dionex ASE 350 System (Thermo Scientific, Waltham, MA, USA). The extraction parameters were automatically controlled through a control panel. Stainless steel cells (100 mL) with a cellulose filter were used; each cell was loaded with 15 g of each type of pomace (dried and previously ground) and diatomaceous earth and the working parameters were as follows: solvent used: ethanol solution (50%, *v*/*v*); temperature: 60 °C; static time: 10 min and number of cycles: 3. The extracts were collected in 200 mL glass vials and stored at 4 °C. According to the ASE extracts’ volume, the concentration of the extracts was 10% (*w*/*v*).

The extracts have been processed through membrane technologies: micro- and ultrafiltration through a KMS Laboratory Cell CF-1 laboratory installation. Microfiltration was performed with microfiltration membranes with a pore size of 0.45 μm, at a pressure of 3–4 bar, and for ultrafiltration membranes a cut-off of 1000 Da was used.

### 3.3. Analytical Methods

#### 3.3.1. Determination of Total Phenolic and Flavonoids Content (TPC and TFC)

The total phenolic content (TPC) of grape and lingonberry pomace extracts was determined according to the Folin–Ciocalteu method [86]. The total phenolic content of the sample was expressed as chlorogenic acid equivalents (CAE) µg/mL using the calibration curve y = 0.0016x + 0.013 with R^2^ = 0.9945.

The total flavonoid content (TFC) in the extracts was evaluated using the aluminum chloride colorimetric method [87]. The results were presented as rutin equivalents (RE) µg/mL extract based on a rutin calibration curve, y = 0.0025x + 0.009 with R^2^ = 0.9987.

#### 3.3.2. HPLC Determination of Individual Phenolic Compounds

A Shimadzu system consisting of two pumps (LC-20AD), a degassing unit (DGU-20A), column oven (CTO-20A), and an autosampler (SIL-20AC), and a photodiode array detector (SPDM20A) was used for HPLC analyses. Absorbance values were recorded with a photodiode array detector (PDA) in the range of 200–600 nm, and the wavelengths of each peak were selectively selected based on the maximum absorbance of each compound.

The separation of phenolic acids and flavonoids was carried out on a Luna Phenomenex C18 column, (250 × 4.6 mm, 10 µm) set at 20 °C during the analysis. The mobile phase was composed of acidified water (0.1% formic acid, pH = 3) as solvent A and methanol/acetonitrile (50:50, *v*/*v*) acidified with formic acid as solvent B. The elution conditions were as follows: 5% solvent B from 0 to 5 min, an increase to 30% solvent B from 5 to 25 min, a decrease to 25% solvent B from 25 to 30 min, then maintaining 25% solvent B from 30 to 38 min, maintaining 30% solvent B from 38.01 to 40 min, an increase to 30–50% solvent B from 40.01 to 57 min, then maintaining 50% solvent B from 57 to 58 min, a decrease to 5% solvent B from 58 to 60 min, and then maintaining 5% solvent B from 60 to 70 min. Also, to make the separation more efficient, a flow rate gradient was used, namely 1 mL min^−1^ from 0 to 5 min, 1.5 mL min^−1^ from 5 to 15 min, 1 mL min^−1^ from 15 to 35 min, 1–1.5 mL min^−1^ from 35 to 40 min, 1.5 mL min^−1^ from 40 to 45 min, 1 mL min^−1^ from 45 to 47 min, 0.75 mL min^−1^ from 47 to 50 min, and 1.5 mL min^−1^ from 50 to 70 min. The PDA was set at 210–600 nm and chromatograms were extracted at 280, 327, and 360 nm for phenolic acids and flavonoids.

Chromatographic separation anthocyanidins and anthocyanins was performed on a Kromasil C18 column (250 × 4.6 mm, 10 µm) at 40 °C, with a mobile phase consisting of water with 5% formic acid (solvent A) and methanol with 5% formic acid (solvent B). The gradient composition was as follows: 6–10% solvent B from 0 to 15 min, 10–50% solvent B from 15 to 35 min, 50–6% solvent B from 35 to 36 min, 6% solvent B from 36 to 45 min; with the flow rate gradient, 1 mL min^−1^ from 0 to 29 min, 0.75 mL min^−1^ from 29 to 33 min, and 1 mL min^−1^ from 33 to 45 min. The DAD detector was set over the range 500–550 nm and the total analysis time was 45 min.

### 3.4. Antioxidant Assays

#### 3.4.1. DPPH Radical Scavenging Activity

The DPPH scavenging assay is based on electron donation of antioxidants to neutralize DPPH radicals. In our study, a previously reported method was used [88], with minor modifications. Briefly, serial diluted solutions of the extract (3 mg/mL, 1.5 mg/mL, 0.75 mg/mL, 0.3 mg/mL) were mixed with methanol and DPPH solution, incubated in the dark for 3 min, and the absorbance was determined at 517 nm against methanol as a blank sample. The radical scavenging activity expressed as percentage of inhibition of DPPH radical was calculated using the following equation:

I (%) = [(A_control_ − A_sample_)/A_control_] × 100

Also, the antioxidant activity by the DPPH scavenging method is often reported as IC_50_ which is defined as the effective concentration of the antioxidant from the extracts necessary to decrease the initial DPPH concentration by 50%. The results for IC_50_ ≤ 50 μg/mL indicate that the antioxidant capacity is good; IC_50_ between 50 and 100 μg/mL shows a mild antioxidant capacity, while IC_50_ ≥ 200 μg/mL point out an insignificant antioxidant capacity [89]. Significant statistical differences were considered *p* < 0.05.

#### 3.4.2. Reducing Power Activity

The reducing power assay is often used to evaluate the ability of the compounds to reduce Fe^3+^ to Fe^2+^ by electron transfer reaction. The assay was performed according to the method described by Berker et al. [90]. For this purpose, a 0.1 mL sample was mixed with 2.5 mL sodium phosphate buffer (200 mM/L, pH 6.6) and 2.5 mL potassium ferricyanide (1%), stirred and heated at 50 °C for 20 min, and after that 2.5 mL trichloroacetic acid (10% *w*/*v*), 2.5 mL deionized water, and 0.5 mL ferric chloride (0.1%) were added. The EC_50_ value (µg/mL) is the extract concentration at an absorbance of 0.5 (a.u.) for reducing power and was calculated from the graph of absorbance at 700 nm against extract concentration.

Significant statistical differences were considered as *p* < 0.05. Ascorbic acid was used as the reference chemical in all assays.

### 3.5. Enzymes Inhibition Activity

In this study, we highlight the importance of exploring new therapeutic alternatives for the treatment of diabetes and obesity, particularly focusing on the inhibition of digestive enzymes by the lingonberry and grape pomace extracts and their bioactive constituents. Acarbose, a known pharmacological inhibitor of α-glucosidase and pancreatic α-amylase, was used as the positive control in the α-glucosidase and α-amylase assays.

#### 3.5.1. Amylase Inhibition Assay

The α-amylase inhibition assay was performed according to Ranilla et al., with some modifications [91]. Briefly, 100 μL extract sample with 250 μL hog pancreas α-amylase (EC 3.2.1.1) (0.5 mg/mL) in sodium phosphate buffer (0.02 M, pH 6.9) was mixed and heated at 37 °C for 20 min; then, 250 μL starch solution (1%) was added, and the mixture was reincubated at 37 °C for 30 min. Afterward, 500 μL of dinitrosalicylic acid (DNS) was added and the reaction mixture was boiled for 5 min. Finally, 5 mL of distilled water was added to the mixture and the absorbance was read at 540 nm with a UV-visible spectrophotometer (Jasco-V630). The inhibition rate was calculated using the equation as follows:

% Amylase inhibition = [(A_control_ − A_sample_)/A_control_] × 100

IC_50_ (extract concentration causing 50% enzyme inhibition) values were determined by linear regression analysis and significant statistical differences were considered as *p* < 0.05.

#### 3.5.2. α-Glucosidase Inhibition

The α-glucosidase inhibitory assay was adapted from Queiroz et al., with minor modification [92]. In brief, 120 μL α-glucosidase (EC 3.2.1.20) (0.5 U/mL) with 720 μL sodium phosphate buffer (0.1 M, pH 6.9) and 60 µL extract sample were mixed and heated at 37 °C, for 15 min. Then, a 120 μL p-nitrophenyl-α-D-glucopyranoside (5 mM/L) solution was added, and the mixture was heated again at 37 °C, for 15 min. Finally, the absorbance was read at 405 nm. The inhibition rate was calculated using the formula:

% Glucosidase inhibition = [(A_control_ − A_sample_)/A_control_] × 100

IC_50_ values were calculated by the linear regression analysis, while significant statistical differences were considered at *p* < 0.05.

### 3.6. Statistical Analysis

Each sample of extracts was analyzed in triplicate. The results were presented using descriptive statistics, which summarize data using indexes such as mean, median, and standard deviation (SD), as mean ± SD. One-way analysis of variance (ANOVA) was used to determine the significant differences, the variability between the means (F and F-critical values, where a larger F-value indicates that the variations between the groups are significant) and the probability (if *p* < 0.05, there are differences among the compared groups). Also, the data were analyzed using Student’s *t*-test and the obtained values were considered good when *p* < 0.05.

## 4. Conclusions

The experimental results highlighted that the type and level of bioactive compounds in the analyzed extracts differed depending on the vegetal material, the method used for processing the plant extract, and the extraction process. All concentrated extracts obtained by membrane technologies presented significantly higher antioxidant and inhibition activities for the studied enzymes compared with the raw extracts, demonstrating the efficiency of these technologies in the recovery and concentration of bioactive compounds from residues and by-products. The grape pomace concentrated extracts obtained by ASE showed higher inhibitory activity than those obtained by UAE, while for the concentrated lingonberry pomace, extracts obtained by UAE showed higher inhibitory activity than those obtained by ASE. The valorization of lingonberry and grape pomace, the by-products from agro-industrial sector, is of utmost importance for the preservation of the environment and for financial purposes. The extracts obtained by two ecofriendly extraction methods provide a wide range of bioactive compounds with great impact on human health, contributing to the prevention of chronic diseases (e.g., diabetes mellitus). Given the growing interest in functional foods and personalized nutrition programs, such research could play an important role in the development of precise dietary supplements aimed at treating chronic diseases based on greener and safer technologies.

## Figures and Tables

**Table 1:** Total phenolic and total flavonoid content in the obtained extracts (values are mean ± SD (n = 3) and ANOVA results).

Samples		Polyphenol Concentration ± SD (CAE μg/mL)		Flavonoid Concentration ± SD (RE μg/mL)	
		UAE	ASE	UAE	ASE
Lingonberry pomace extracts	Microfiltrate	1277.50 ± 15.67	1804.37 ± 24.36	219.50 ± 8.58	336.25 ± 12.31
	Concentrate	1653.75 ± 27.89	2440.62 ± 14.56	287.75 ± 6.32	495.65 ± 22.52
F		*p*-value		F crit	
34.25		0.001		5.98	
Grape pomace extracts	Microfiltrate	744.67 ± 15.32	446.93 ± 11.23	81.20 ± 2.58	55.10 ± 2.13
	Concentrate	1041.12 ± 26.25	571.51 ± 10.56	124.40 ± 5.62	70.65 ± 1.35
F		*p*-value		F crit	
22.75		0.003		5.98	

CAE = chlorogenic acid equivalent; RE = rutin equivalent; *p*-value = the probability value; F and F-critical values = the variability.

**Table 2:** Polyphenolic profile for lingonberry residues extracts (values are mean ± SD (n = 3) and ANOVA results).

Compound	Extract UAE		Extract ASE	
	Initial Concentration ± SD	Concentrate Concentration ± SD	Initial Concentration ± SD	Concentrate Concentration ± SD
	μg/mL	μg/mL	μg/mL	μg/mL
Gallic acid	4.88 ± 0.23	5.14 ± 0.25	6.86 ± 0.42	8.89 ± 0.42
(+)-Catechin	241.26 ± 12.56	279.39 ± 18.6	328.27 ± 21.06	331.66 ± 15.63
Chlorogenic acid	60.51 ± 3.56	71.79 ± 4.89	47.98 ± 3.21	89.09 ± 4.65
Caffeic acid	13.05 ± 0.89	13.02 ± 0.78	21.47 ± 1.76	27.76 ± 1.89
(−)-Epicatechin	22.01 ± 1.89	29.35 ± 1.32	21.89 ± 1.78	43.39 ± 2.56
p-Coumaric acid	2.21 ± 0.15	2.58 ± 0.19	2.63 ± 0.12	5.64 ± 0.39
Rutin	7.32 ±0.56	8.56 ± 0.46	13.73 ± 0.89	22.19 ± 1.56
Ellagic acid	7.32 ± 0.32	5.70 ± 0.23	12.85 ± 0.89	21.91 ± 1.24
Quercetin 3-β-D-glucoside	6.49 ± 0.37	7.47 ± 0.35	10.93 ± 0.75	16.52 ± 0.89
Quercitrin	14.06 ± 0.7	5.70 ± 0.31	18.28 ± 0.95	44.16 ± 2.56
Myricetin	4.67 ± 0.23	5.70 ± 0.25	3.15 ± 0.12	7.04 ± 0.35
Quercetin	5.70 ± 0.21	5.93 ± 0.12	7.91 ± 0.51	8.89 ± 0.56
Luteolin	-	-	-	-
Kaempferol	0.44 ± 0.02	0.46 ± 0.01	0.54 ± 0.02	0.53 ± 0.03
Isorhamnetin	-	-	-	-
F	*p*-value		F crit	
119.98	6.8 × 10^−27^		2.01	
Delphinidin-3-glucoside	75.85 ± 2.36	84.57 ± 3.56	69.03 ± 2.36	73.22 ± 5.63
Cyanidin-3-glucoside	74.34 ± 3.52	74.56 ± 2.89	55.13 ± 2.14	65.33 ± 3.56
Petunidin-3-glucoside	1.28 ± 0.06	1.58 ± 0.56	0.77 ± 0.02	0.88 ± 0.05
Peonidin-3-glucoside	1.16 ± 0.03	1.24 ± 0.38	1.19 ± 0.05	1.08 ± 0.07
Malvidin-3-glucoside	0.78 ± 0.04	1.07 ± 0.26	0.16 ± 0.01	0.59 ± 0.03
Delphinidin	0.42 ± 0.01	0.46 ± 0.02	0.39 ± 0.02	0.37 ± 0.01
Cyanidin	0.53 ± 0.02	0.55 ± 0.03	0.44 ± 0.01	0.48 ± 0.01
F	*p*-value		F crit	
261.54	1.3 × 10^−18^		2.57	

**Table 3:** Polyphenolic profile for grape pomace extracts (values are mean ± SD (n = 3) and ANOVA results).

Compound	Extract UAE		Extract ASE	
	Initial Concentration ± SD	Concentrate Concentration ± SD	Initial Concentration ± SD	Concentrate Concentration ± SD
	μg/mL	μg/mL	μg/mL	μg/mL
Gallic acid	3.59 ± 0.12	4.93 ± 0.25	4.83 ± 0.14	4.96 ± 0.02
(+)-Catechin	7.38 ± 0.34	13.35 ± 0.56	10.78 ± 0.3	13.20 ± 0.85
Chlorogenic acid	0.97 ± 0.02	2.72 ± 0.12	0.52 ± 0.02	0.75 ± 0.04
Caffeic acid	0.56 ± 0.03	1.24 ± 0.05	0.46 ± 0.02	0.51 ± 0.02
(−)-Epicatechin	10.72 ± 0.75	13.97 ± 0.29	21.02 ± 1.36	22.59 ± 1.35
p-Coumaric acid	-	-	-	-
Rutin	4.93 ± 0.02	6.18 ± 0.23	1.73 ± 0.07	1.12 ± 0.08
Ellagic acid	1.88 ± 0.04	2.35 ± 0.06	0.54 ± 0.02	0.54 ± 0.03
Quercetin 3-β-D-glucoside	5.60 ± 0.28	6.25 ± 0.34	3.96 ± 0.13	3.91 ± 0.18
Quercitrin	5.18 ± 0.32	12.88 ± 0.89	7.29 ± 0.36	9.69 ± 0.57
Myricetin	0.27 ± 0.01	0.60 ± 0.02	0.77 ± 0.05	1.05 ± 0.06
Quercetin	1.62 ± 0.02	2.34 ± 0.07	0.90 ± 0.06	0.76 ± 0.05
Luteolin	0.55 ± 0.01	0.17 ± 0.01	0.35 ± 0.02	0.36 ± 0.02
Kaempferol	0.36 ± 0.02	0.50 ± 0.02	0.33 ± 0.01	0.32 ± 0.01
Isorhamnetin	0.21 ± 0.01	0.18 ± 0.01	0.13 ± 0.01	0.11 ± 0.01
F	*p*-value		F crit	
23.29	4.5 × 10^−15^		1.96	
Delphinidin-3-glucoside	0.88 ± 0.04	1.18 ± 0.08	1.46 ± 0.58	3.03 ± 0.02
Cyanidin-3-glucoside	0.74 ± 0.04	1.23 ± 0.07	1.70 ± 0.06	2.25 ± 0.18
Petunidin-3-glucoside	0.53 ± 0.03	0.39 ± 0.02	0.76 ± 0.05	0.76 ± 0.05
Peonidin-3-glucoside	1.77 ± 0.06	1.59 ± 0.01	1.44 ± 0.06	1.65 ± 0.01
Malvidin-3-glucoside	19.79 ± 0.89	21.76 ± 1.56	18.97 ± 0.69	20.36 ± 1.56
Delphinidin	-	-	-	-
Cyanidin	0.43 ± 0.02	0.41 ± 0.03	0.50 ± 0.02	0.84 ± 0.05
Malvidin	0.48 ± 0.01	0.42 ± 0.02	0.55 ± 0.04	0.67 ± 0.03
F	*p*-value		F crit	
523.11	9.7 × 10^−23^		2.57	

**Table 4:** Antioxidant activity of lingonberry and grape pomace extracts.

Samples		DPPH Radical Scavenging Activity IC_50_ (µg/mL)		Reducing Power Activity EC_50_ (µg/mL)	
		UAE	ASE	UAE	ASE
Lingonberry pomace extracts	Microfiltrate	1.24 ± 0.02 *	1.17 ± 0.08 *	61.83 ± 2.35 **	78.60 ± 3.15 **
	Concentrate	0.67± 0.01 *	0.69 ± 0.02 *	50.02 ± 1.56 **	50.52 ± 1.25 **
Grape pomace extracts	Microfiltrate	1.60 ± 0.05 *	1.39 ± 0.03 *	86.81 ± 4.52 **	67.51 ± 3.23 **
	Concentrate	0.46 ± 0.02 *	0.30 ± 0.01 *	60.45 ± 2.13 **	39.39 ± 1.56 **
Vitamin C		1.18 ± 0.02		42.47 ± 1.32	

Experiments were performed in triplicate; * *p* < 0.05, radical scavenging activity comparative with the polyphenols and flavonoids content of the extracts. ** *p* < 0.05, reducing power compared with the polyphenols and flavonoids content of the extracts.

**Table 5:** α-amylase and α-glucosidase inhibition activity of the investigated extracts.

Samples		α-Amylase Inhibition IC_50_ (µg/mL)		α-Glucosidase Inhibition IC_50_ (µg/mL)	
		UAE	ASE	UAE	ASE
Lingonberry pomace extracts	Microfiltrate	3.67 ± 0.12 *	4.75 ± 0.15 *	2.92 ± 0.12 *	2.84 ± 0.13 *
	Concentrate	1.64 ± 0.09 *	3.49 ± 0.21 *	1.46 ± 0.06 *	2.32 ± 0.15 *
Grape pomace extracts	Microfiltrate	1.84 ± 0.08 *	0.98 ± 0.02 *	1.58 ± 0.08 *	0.93 ± 0.02 *
	Concentrate	0.69 ± 0.01 *	0.30 ± 0.01 *	1.12 ± 0.07 *	0.60 ± 0.01 *
Acarbose		0.30 ± 0.01		0.57 ± 0.02	

The data represent the average of experiments performed in triplicate; * *p* < 0.05, the α-amylase inhibition activity comparative with the polyphenols and flavonoids content of the extracts; * *p* < 0.05, the α-glucosidase inhibition comparative with the polyphenols and flavonoids content of the extracts.

## Data Availability

Data are contained within the article.
